# 4393 Translational Characterization of Blood Pressure Changes Following the DASH Diet– from Nutrition to Electrolytes to Exosomes

**DOI:** 10.1017/cts.2020.157

**Published:** 2020-07-29

**Authors:** Dana Bielopolski, Andrea Ronning, Dacia Vasquez, Glenis George-Alexander, Jeanne Walker, Jonathan N. Tobin, Rhonda G Kost

**Affiliations:** 1 Rockefeller University

## Abstract

OBJECTIVES/GOALS:

1. analyze urinary protein exosome content pattern before and during DASH diet.
2. characterize urine electrolyte changes associated with changes in protein profiles, and hormonal changes before/after DASH diet.
3. analyze the association of these changes to the DASH-related BP response.

METHODS/STUDY POPULATION: In this proof of concept study, hypertension stage 1 volunteers will receive a DASH based menu during 14 consecutive days of elective admission to the RU research hospital. Participants will complete a food frequency questionnaire (VioScreen) with a bionutritionist. Throughout the intervention period, participants will be assessed for blood pressure, plasma renin and aldosterone, and 24 hour urines for electrolytes, creatinine, protein, albumin and first morning urine collected for exosomes. Exosome analysis will be performed by a commercial lab. Proteome analysis will be conducted in the RU Mass-spectrometry service. RESULTS/ANTICIPATED RESULTS: The causal pathway we will elucidate hypothesizes that: 1) changes in diet affect blood electrolytes, and through these, aldosterone. 2) Aldosterone alters the expression of specific transporter proteins in the renal tubule; protein expression will be reflected in the urine exosome. 3) These transporters affect the excretion of electrolytes, as reflected by urinary ratio of sodium (Na) to Potassium (K). During consumption of the Western diet, the Na/K ratio is approximately 2-2.5, whereas we expect the urinary sodium/potassium ratio to be <1, when the participant is eating a DASH based diet. DISCUSSION/SIGNIFICANCE OF IMPACT: This assay provides a clinical tool to assess dietary adherence, and the project will provide insights into the mechanism whereby DASH reduces blood pressure.

